# Making State Public Health Laws Work for SARS Outbreaks

**DOI:** 10.3201/eid1002.030836

**Published:** 2004-02

**Authors:** Edward P. Richards, Katharine C. Rathbun

**Affiliations:** * Louisiana State University Law Center, Baton Rouge, Louisiana, USA; † Ochsner Clinic Foundation, Baton Rouge, Louisiana, USA

**Keywords:** law, legal aspects, lawyers, constitutional law, quarantine, patient isolation

In their article, HHS/CDC Legal Response to Outbreak of Severe Acute Respiratory Syndrome [SARS], Misrahi, et al. (*1*) describe the updated federal laws and response plans for handling SARS and related communicable diseases. Federal authority is important to control the interstate and international movement of persons who are potentially infectious, but most isolation and quarantine orders will be performed by state and local officials, using state and local law. We discuss how existing laws might be modified to facilitate effective SARS control while providing legal protections to restricted persons.

## Traditional Powers

The drafters of the U.S. Constitution gave states broad powers to control communicable diseases because the colonies were ridden with malaria, yellow fever (*2*), cholera, and typhoid. States exercised these powers as necessary, quarantining persons and even whole cities and regions (*3*). This public health authority has been upheld by the U.S. Supreme Court in all cases (*4*), except when it is was clearly a subterfuge for racial discrimination (*5*), and in 1950, every state and local health department had clear powers to conduct case-finding and isolate or quarantine persons who represented a potential public health risk (*6*).

State public health laws do not need to be detailed and specific, but they can give public health agencies the general authority to protect the public’s health and safety. Consistent with the Constitution, courts allow government agencies to fill in the details of these laws (*7*). Statutes do not need specific judicial review because all detentions are reviewable through habeas corpus proceedings. Habeas corpus is a fundamental part of Anglo-American law, protecting persons against illegal detention. A part of the U.S. Constitution, habeas corpus needs no additional statutory authorization, although all states provide for it.

Persons detained by the state may file a habeas corpus petition and demand that a court review their detention. In the case of quarantine due to disease, a judge would determine whether the state has shown that the detained person deserves quarantine. The judge must defer to public health authorities on their choice of public health strategies (*8*). Public health orders get the most permissive judicial review, the rational relationship test, because they are based on objective criteria, are usually of limited duration, and are necessary to prevent imminent harm (*9*).

## Contemporary Public Health Laws

With the advent of AIDS in the 1980s, some civil libertarians argued that the old public health laws were outdated and no longer enforceable. There was no judicial support for this argument then (*10*), and today’s courts are even more supportive of state powers to protect the public. Nonetheless, many states rewrote their isolation and quarantine laws to provide varying levels of mandatory judicial review, in some cases requiring that a person be provided counsel and an opportunity for a trial before detention. Such proceedings take so much time and money that they make it almost impossible to impose quarantine (*11*).

Even public health laws rewritten in the wake of the 9/11 events often include judicial review provisions that would be unworkable in a large outbreak; persons would either be detained illegally or be released because of legal technicalities. Improperly detained persons can sue, and these lawsuits will probably not be barred by the immunity provisions in emergency public health laws. Improperly released persons will nullify the disease control plan.

## Administrative Law Solution

The best way to balance public protection with private rights is to use administrative hearings rather than judicial hearings to review quarantine and other public health orders. Administrative review is used routinely in state and federal agency proceedings, including for mental health commitments in Maryland (*12*). Courts have required more due process for mental health commitments than for quarantines; this difference is strong evidence that administrative review would be an acceptable alternative for public health orders. Such reviews can be appealed to the courts, but having the agency do the first review makes a factual record that allows quick and efficient judicial review. A petitioner can be required to go through an agency appeal before a habeas corpus review by the courts (*13*).

Persons who want to contest their isolation orders could be required to petition the decision maker doing the reviews. This petition could be to a health agency staff member or an appointed board. The health agency would present the basic information, and the petitioner could supply additional information in writing. Telephone interviews could be used to allow personal statements without the danger of in-person testimony. The decision maker would make a brief, written ruling based on predefined classifications. This ruling could be reviewed by an agency appeals board and would greatly simplify any subsequent appeal to the courts (*14*). If such a process is adopted, the statutory language to implement these reviews should be kept general to allow flexibility in the face of different epidemic conditions.

Such a review should also be part of the quality assurance for isolation and quarantine orders. A key part of any isolation and quarantine process for SARS would be thorough recordkeeping of all orders, whom such orders apply to, their duration, and the disease outcome in each case. There should be administrative oversight to ensure that the orders are proper and that other necessary actions are carried out, such as providing food and medical services to restricted persons.

## Conclusions

A major SARS outbreak would stretch many state and local public health laws to the breaking point. These laws should be reviewed and rewritten as necessary. Fair process can be based on sound administrative law principles that dramatically reduce the role of judicial review in isolation and quarantine orders.
